# Deregulated *Nras* Expression in Knock-In Animals Harboring a Gammaretroviral Long Terminal Repeat at the *Nras*/*Csde1* Locus

**DOI:** 10.1371/journal.pone.0056029

**Published:** 2013-02-13

**Authors:** Borja Ballarín-González, Louise Berkhoudt Lassen, Randi Jessen, Annette Füchtbauer, Ernst-Martin Füchtbauer, Finn Skou Pedersen

**Affiliations:** Department of Molecular Biology and Genetics, Aarhus University, Aarhus, Denmark; National Institute of Allergy and Infectious Diseases, United States of America

## Abstract

To investigate mechanisms and phenotypic effects of insertional mutagenesis by gammaretroviruses, we have developed mouse lines containing a single Akv 1-99 long terminal repeat (LTR) and a floxed PGK/Tn5 neomycin cassette at the *Nras* proto-oncogene at positions previously identified as viral integration sites in Akv 1-99 induced tumors. The insert did not compromise the embryonic development, however, the cassette had an effect on *Nras* expression in all tissues analyzed. Cre-mediated excision of the PGK/Tn5 neomycin cassette in two of the lines caused upregulation of *Nras*. Altogether, the knock-in alleles are characterized by modulation of expression of the target gene from more than ten-fold upregulation to three-fold downregulation and exemplify various mechanisms of deregulation by insertional mutagenesis. LTR knock-in mice may serve as a tool to investigate mechanisms of retroviral insertional mutagenesis and as a way of constitutive or induced modulation of expression of a target gene.

## Introduction

Retroviruses insert a double-stranded DNA copy of their genome non-specifically into the genome of the host and thereby act as insertional mutagens that can disrupt gene regulation or cause the production of an altered gene product [1]. The integrated retrovirus, termed the provirus, contains strong transcription-regulatory signals that can induce or enhance the expression of nearby genes. When such affected genes are involved in cell survival and proliferation, their deregulated expression may contribute to tumorigenesis [2]. In tumors caused by retroviral insertions, the proviruses constitute a tag allowing the identification of candidate genes with a possible role in tumorigenesis. By this approach several recent studies have contributed to the discovery of new proto-oncogenes and also, when performed in genetically modified mice expanded our knowledge on oncogene cooperativity [3]–[4]. Moreover, insertional mutagenesis is a concern in gene therapy by retroviral vectors [5]. By another development, evidence is also emerging that endogenous retroviruses of mice and humans may contribute to oncogenesis by the activation of nearby genes without any need for new retroviral insertions in somatic cells [6].

Based on the analysis of somatic integrations selected during malignant transformation various types of virus-induced gene activation have been proposed. By the process known as enhancer insertion a provirus increases the production of a normal transcript of an adjacent target gene [2]. In these cases, proviruses are often found outside the transcription unit of the target gene and in many cases upstream of the target gene and in the opposite transcriptional orientation. Other types of insertional mutagenesis result in the formation of chimeric RNA species containing viral and host sequences. One example of this is promoter insertion in which proviruses are integrated in the same transcriptional orientation as the proto-oncogene, either upstream or within its 5′end [2].

The work reported by Martin-Hernandez et al. [7] represents an example of gene over-expression caused by promoter insertion. Three out of 13 murine B-cell lymphomas induced by the leukemogenic Akv1-99 virus had retroviral integrations into the *Nras*/*Csde1* locus [7]. In all three cases viral-*Nras* chimeric RNAs were detected and the overall level of mRNA with NRAS-encoding potential significantly increased, whereas the retroviral integrations did not influence the expression of *Csde1*. Since no activating mutations of *Nras* were detected, the sole over-expression of the wild type gene seems to constitute an important factor in the development of B-cell lymphomas in this experimental setting.

To further investigate the processes of deregulation by an integrated gammaretrovirus and to assess if intrinsic over-expression of the *Nras* proto-oncogene may be sufficient to induce neoplastic pathologies, we have developed the first target-specific mouse knock-in models using retroviral sequences. These animals contain a single Akv1-99 LTR integrated at the exact same position as a previously identified retroviral integration in a tumor, placed in either the same or opposite transcriptional orientation relative to *Nras* and with or without a flanking floxed PGK/Tn5 neo cassette. We here report that the different alleles up or down-regulate *Nras* expression to various degrees dependent upon the orientation and position of the LTR. Mice of this series have already proven valuable in the analysis of mechanisms of deregulation of host genes by insertional mutagenesis [8] as well as investigation of phenotypic effects of *Nras* over-expression [9].

## Results

### Generation of Alleles with Targeted Knock-in of an LTR and a Floxed PGK/Tn5 Neomycin Cassette

**Figure 1:**
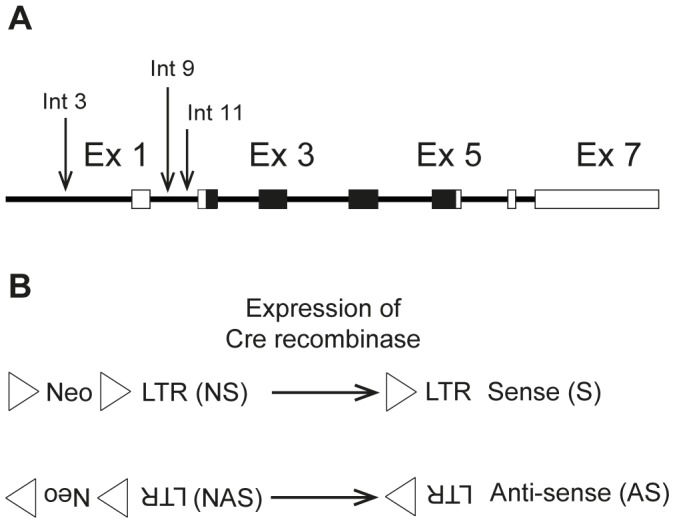
Overview of knock-in alleles. (A). Schematic representation of *Nras*. Arrows indicate the identified Akv 1­99 proviral integrations (integration 3, 9 and 11) [7]. Boxes represent exons and the coding region is depicted in black. (B). Representation of the “targeting cassettes” introduced in the sense (S) and antisense (AS) knock-in models. Upon expression of Cre recombinase a LoxP sequence (triangle) and the neomycin selection marker (Neo) can be removed from the construct. LTR = long terminal repeat.

The positions chosen for targeted insertion of an LTR-containing cassette corresponded to the three retroviral integrations identified in B-cell lymphomas by Martin-Hernandez et al. [7] within an 800 bp window upstream of the coding region for NRAS (Figure 1A). Integration 3 was located in the 3′untranslated region of *Csde1* upstream of the *Nras* promoter, whereas integrations 9 and 11 were both located in intron 1 of *Nras*. All three integrations had the same transcriptional orientation as *Nras*. To address the role of the orientation of the LTR, targeted insertions were made with the LTR in the same as well as the opposite transcriptional orientation as *Nras*. The knock-in plasmids harbored an Akv1-99 LTR and, to allow selection, a floxed PGK-neomycin-resistance expression cassette placed in the same transcriptional orientation as the LTR. The alleles with the neomycin selection marker (neo) and an LTR in sense orientation relative to *Nras* are termed LTR3NS, LTR9NS, and LTR11NS for the three positions, respectively, whereas the alleles with neo and an LTR in antisense orientation relative to *Nras* are termed LTR3NAS, LTR9NAS, and LTR11NAS (Figure 1B). As seen in Figure 1B, neo was placed upstream of the LTR relative to the transcriptional orientation of *Nras* in all cases. G418 resistant colonies of CJ7 ES cells [10] with the desired inserts were identified by Southern blotting.

### The LTR Knock-in Cassette Affects Nras Expression in ES Cells

**Figure 2:**
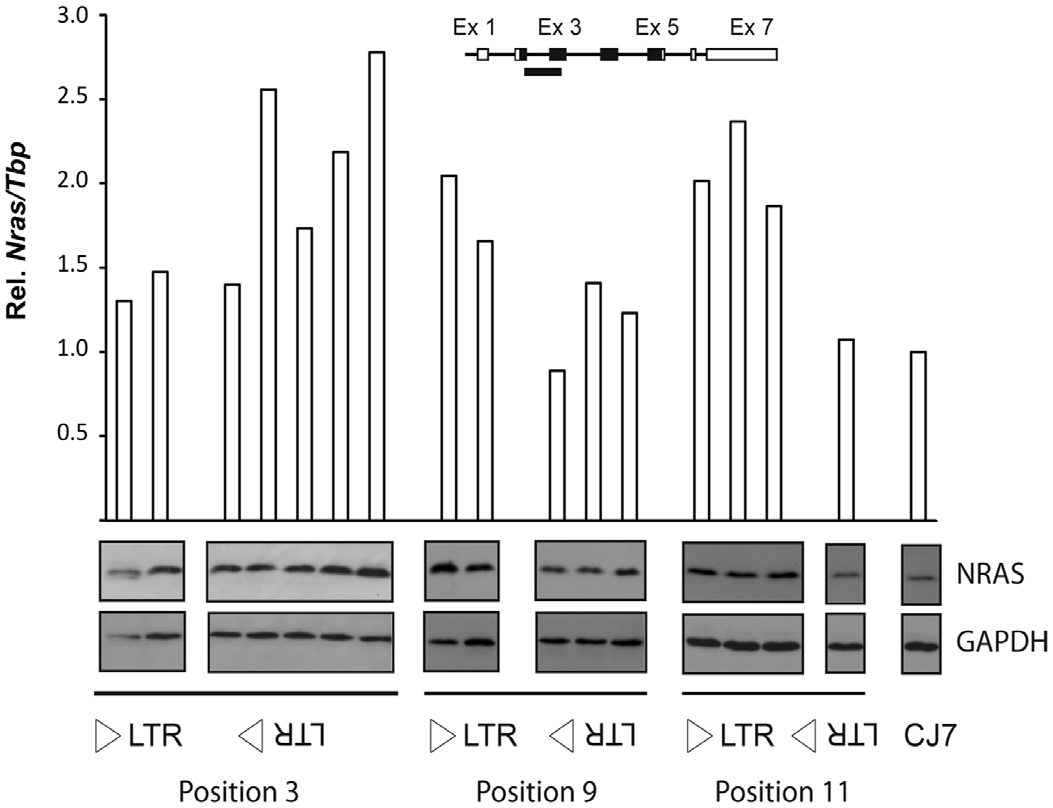
Analysis of *Nras* expression in knock-in clones of mouse ES cells. The analysis included two LTR3NS clones, five LTR3NAS clones, two LTR9NS clones, three LTR9NAS clones, three LTR11NS clones, one LTR11NAS clones as well as parental CJ7 cells. *Nras* mRNA was quantified by qPCR employing an amplicon covering part of exon 2 and exon 3 (insert). Expression was normalized to that of *Tbp* and represented as relative to the parental CJ7 ES cell line. The panels below the histogram present Western blot analysis of NRAS in protein extracts from the listed ES cell clones. GAPDH was used as reference.

To address the effect of the modified alleles on *Nras* expression, quantitative real-time PCR (qPCR) analysis was done using an amplicon spanning the exon 2-exon 3 junction of *Nras*. Analysis of the CJ7-derived clones (Figure 2) showed that the position 3 knock-in alleles had only a minor effect in sense orientation and a pronounced effect in antisense orientation in four out of five clones analyzed. On the other hand, for positions 9 and 11, the CJ7-derived clones showed a pronounced upregulation of *Nras* for knock-in alleles in sense orientation and only a minor effect in case of anti-sense orientated alleles. Western blotting analysis using an NRAS-specific antibody confirmed that the knock-in alleles also had an effect on the levels of NRAS (Figure 2).

### Nras Transcription is Deregulated in Animals with a Cassette in Intron 1

**Figure 3:**
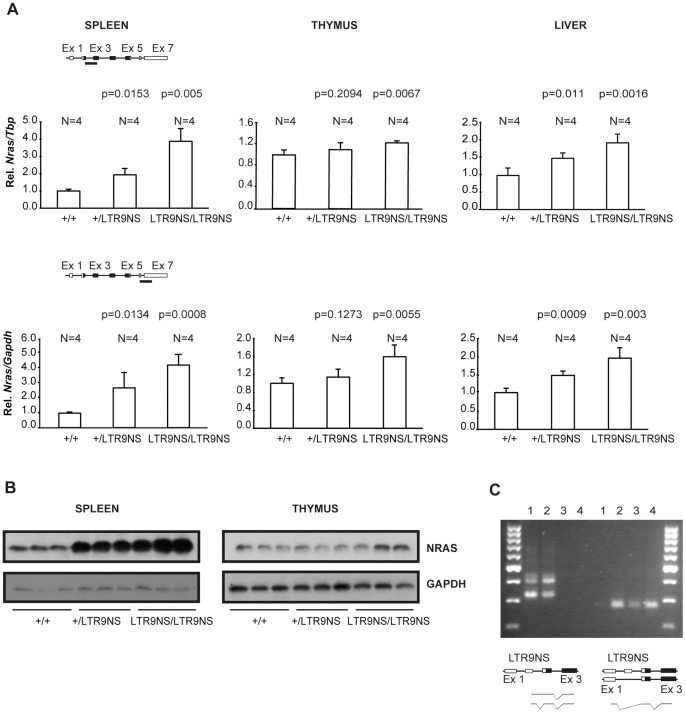
Analysis of knock-in animals harboring the LTR integrated in the sense orientation at position 9. (A). *Nras* expression was quantified by qPCR employing two different methods, SYBR green (amplicon covering part of exon 2 and 3) or a TaqMan hydrolysis probe (amplicon covering part of exon 6 and 7). Expression was normalized to that of *Tbp* or *Gapdh* depending on the employed strategy (SYBR green or TaqMan probe, respectively) and represented as relative to that of wild type animals. N represents the number of animals in the different groups. Paired Student’s t test was used to determine p-values relative to +/+ animals. (B). Western blot analyses of spleen and thymus samples using antibodies against NRAS or GAPDH. C) PCR analysis of mRNA from spleen of homozygous LTR9NS (samples 1 and 2) and wild type animals (samples 3 and 4). Two distinct chimeric mRNAs can be detected by an LTR and an *Nras* specific primer in combination (left half of gel). These transcripts depicted at the bottom of the figure contain viral as well as cellular sequences and differ in length due to splicing or not from a cellular splice donor at the first *Nras* intron. LTR initiated transcription does not seem to suppress the activity of the normal *Nras* promoter, as the putative *Nras* transcript could be detected in both wild type and homozygous LTR9NS animals employing the appropriate *Nras* specific primers (right half of gel).

The effect of the knock-in alleles was first analyzed in animals targeted in intron 1 using position 9 as the example. Mice heterozygous or homozygous for the two position 9 alleles, LTR9NS and LTR9NAS, were both born at the expected ratios and phenotypically normal. To assess the influence of the knock-in cassettes on *Nras* transcription, we employed qPCR using two amplicons covering parts of exon 2 and exon 3 and parts of exon 6 and exon 7, respectively. Introduction of the targeting cassette with the LTR in the same orientation as the *Nras* gene (the LTR9NS allele) caused a clear increase of *Nras* mRNA levels in spleen, thymus and liver (Figure 3A). The measured increase in mRNA levels was similar for the two amplicons. In all cases the heterozygous +/LTR9NS animals had *Nras* mRNA levels between the wild type (+/+) and homozygous knock-in (LTR9NS/LTR9NS) animals. The effect on *Nras* mRNA levels was highest in the spleen, where homozygous knock-in animals showed a four-fold increase in *Nras* mRNA relative to wild type (wt).

Western blotting using an NRAS specific antibody detected higher protein levels in knock-in than in wt animals, again more pronounced in spleen than in thymus (Figure 3B). The liver samples were excluded from the Western analysis due to a low signal to noise ratio.

Expression of the LTR-*Nras* chimeric transcripts previously identified in the tumor harboring a provirus at position 9 [7] was verified by the RT-PCR using LTR and *Nras* specific primers, and it was confirmed that the generation of these transcripts does not abolish transcription from the normal *Nras* promoter (Figure 3C).

**Figure 4:**
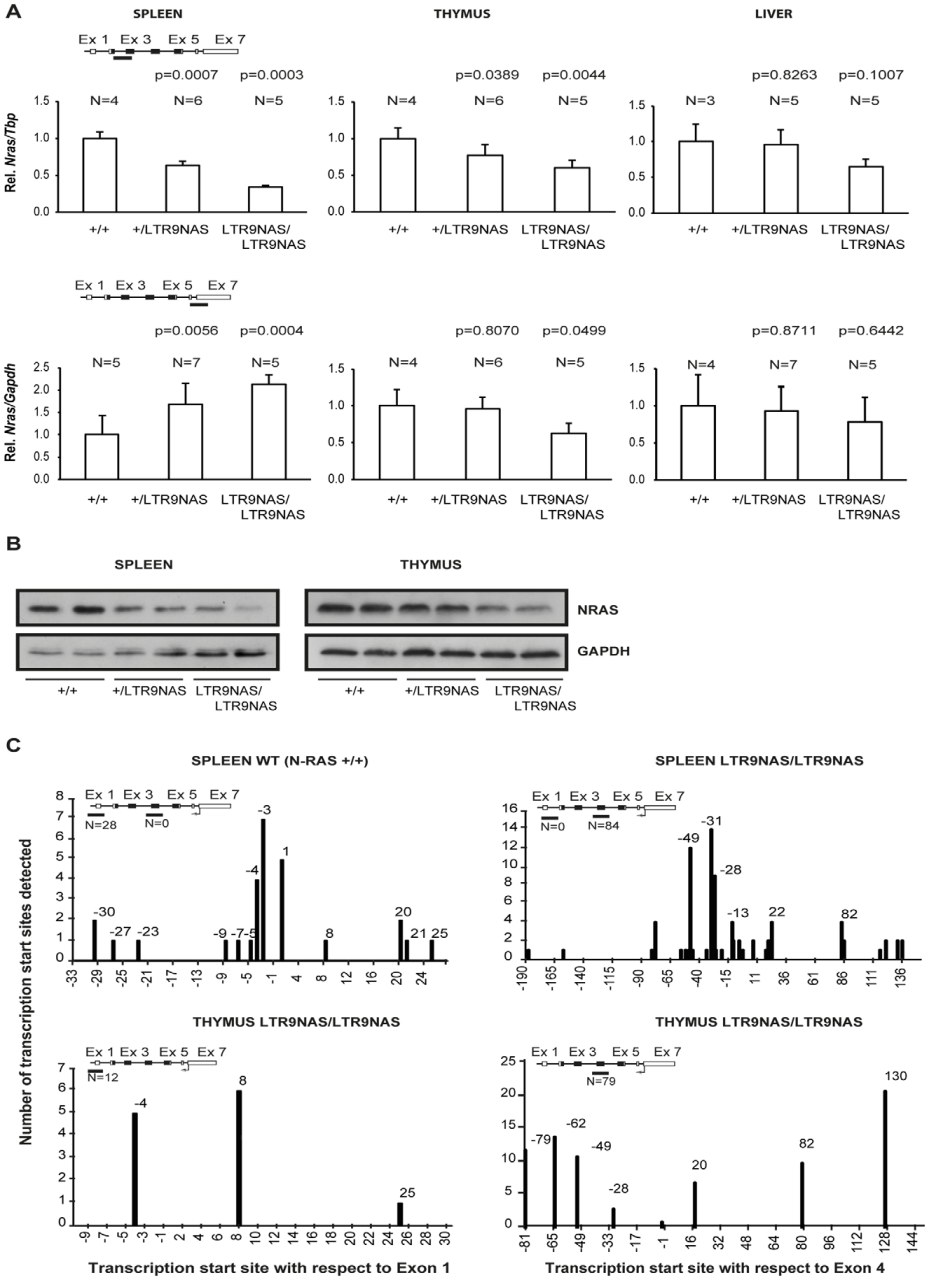
Analysis of knock-in animals harboring the LTR integrated in the antisense orientation at position 9. (A). *Nras* expression was quantified by qPCR employing two different methods, SYBR green (amplicon covering part of exon 2 and 3) or a TaqMan hydrolysis probe (amplicon covering part of exon 6 and 7). Expression was normalized to that of *Tbp* or *Gapdh* depending on the employed strategy (SYBR green or TaqMan probe, respectively) and represented as relative to that of wild type animals. N represents number of animals in the different groups. Paired Student’s t test was used to determine p-values relative to +/+ animals. (B). Western blot analyses of spleen and thymus samples using antibodies against NRAS or GAPDH. (C). Rapid amplification of cDNA ends: Initiation sites of alternative transcripts within the *Nras* gene or viral LTR were identified by the usage of the GeneRacerTM kit (Invitrogen). Position of the detected transcription start sites are depicted with respect to the first nucleotide of exon 1 or 4. Height of the bars indicates the frequency of the detected transcripts.

Analysis of *Nras* mRNA levels in mice harboring the LTR9NAS allele with the LTR placed in the opposite transcriptional orientation of *Nras* revealed downregulation of *Nras* mRNA in spleen, thymus, and liver when analyzed with the amplicon spanning exons 2 and 3 (Figure 4a, upper panels). The mRNA levels in heterozygotes were intermediate between those of wt and of LTR9NAS/LTR9NAS animals. The largest effect was an about two-fold reduction observed in spleen tissue. In contrast, analysis using the amplicon spanning exons 6 and 7 detected an upregulation in animals harboring the LTR9NAS allele in spleen, but not in thymus and liver tissues. Western blotting analysis (Figure 4B) showed a decrease in NRAS protein in mice harboring the LTR9NAS allele in spleen and thymus in consistency with the mRNA levels detected with the exon2-exon3 probe.

The discrepancy between the RNA levels detected in spleen using the two different qPCR probes indicated that alternative RNA species might be induced in the LTR9NAS allele. One of the possible explanations could be the formation of RNA from transcription initiation sites downstream of exon 3. To investigate this, 5′ RACE analysis of *Nras* RNA was done on samples from +/+ and LTR9NAS/LTR9NAS mice (Figure 4C).

As expected, in wild type spleen, all the detected RNA species (28) clustered around the canonical transcription start site for *Nras* mRNA. On the other hand, when spleen from knock-in homozygotes animals was analyzed, a unique cluster of 84 initiation sites was identified at the intron 3/exon 4 boundary. Hence, transcriptional initiation around the intron 3/exon 4 boundary may contribute to the discrepancy between the qPCR data from LTR9NAS/LTR9NAS spleens using the two different qPCR amplicons. The failure to detect the canonical transcription start site most probably results from the selection during the process for the identification of short RNA species and the high expression of these alternative transcripts. The over-representation of these alternative transcripts in 5`RACE analysis was confirmed through the investigation of an LTR9NAS/LTR9NAS thymus. In this tissue, where the same tendency in *Nras* mRNA expression could be observed irrespectively of the utilized qPCR amplicon (Figure 4A), more RNA 5′ends were detected at the alternative than at the canonical promoter. We previously reported that the LTR9NAS allele also expresses *Nras* RNA species initiated at an antisense promoter in the LTR [8] and containing exons 2 and 3 of *Nras*. These data indicate that in LTR9NAS/LTR9NAS animals, *Nras* transcription is deregulated, quantitatively with respect to RNA levels and qualitatively with respect to transcriptional initiation sites.

### Removal of the PGK/Tn5 Neomycin Cassette Leads to More Pronounced Deregulation of Nras Expression

**Figure 5:**
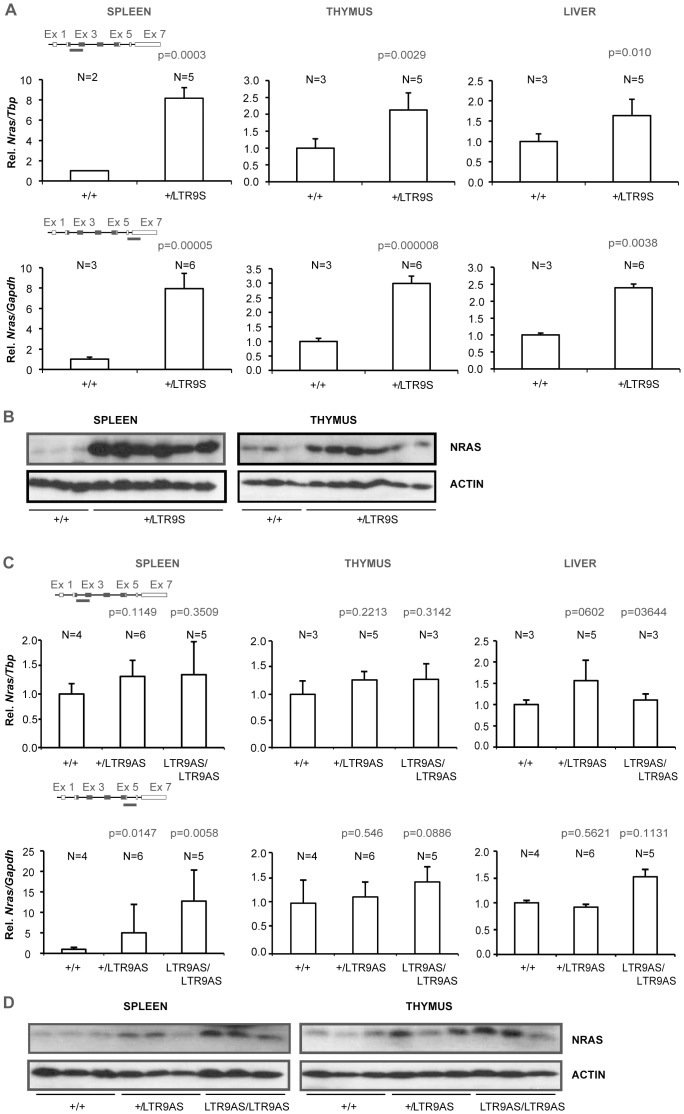
*Nras* expression in knock­in animals with and without the neomycin selection marker. *Nras* expression was quantified by qPCR employing two different methods, SYBR green (amplicon covering part of exon 2 and 3) or a TaqMan hydrolysis probe (amplicon covering part of exon 6 and 7). Expression was normalized to that of *Tbp* or *Gapdh* depending on the employed strategy (SYBR green or TaqMan probe, respectively) and represented as relative to that of wild type animals. Panels A and B: qPCR and Western analysis of the LTR9S allele. Only +/+ and +/LTR9S animals are included since LTR9S/LTR9S animal die within three weeks. Panels C and D: qPCR and Western Blot analysis of the LTR9AS allele. Paired Student’s t test was used to determine p-values relative to +/+ animals.

We next wanted to investigate the effect of removal of the floxed PGK/Tn5 neomycin cassette. Mice harboring the LTR9NS or LTR9NAS alleles were mated with EIIa-Cre transgenic mice and the loss of the floxed cassette verified by PCR. This generated the alleles LTR9S and LTR9AS. *Nras* mRNA levels were measured using the same qPCR amplicons as used in Figures 3 and 4. In spleen, +/LTR9S heterozygotes showed about eight fold higher levels than +/+ animals (Figure 5A). The levels of Nras mRNA in adult LTR9S/LTR9S homozygotes could not be analyzed since these animals had an early lethality phenotype [9]. The results show that LTR9S causes higher *Nras* mRNA levels than LTR9NS in thymus, liver, and spleen. Altogether, the results demonstrate that removal of the PGK/Tn5 neomycin cassette from the allele with the LTR in sense orientation leads to upregulation of *Nras* mRNA, possibly because the LTR and the *Nras* promoter are brought in closer proximity and/or the loss of an inhibitory effect on transcription caused by the neomycin cassette [11]. The Western blot analyses of NRAS protein levels reveal strong upregulation in heterozygous animals relative to wt in agreement with the mRNA levels (Figure 5B).

Comparing mouse strains with alleles LTR9NAS and LTR9AS revealed that removal of the PGK/Tn5 neomycin cassette caused either an upregulation or had no effect on *Nras* mRNA levels. Using the amplicon spanning exons 2 and 3, animals carrying the LTR9AS allele gave higher *Nras* mRNA values than +/+ in spleen and thymus (Figure 5C). The levels detected with the exon 6-exon 7 amplicon were strongly increased in spleen, presumably caused by intragenic transcriptional initiation as observed for the LTR9NAS allele. Western blotting analysis showed that excision of the PGK/neo cassette also caused upregulation at the protein level (Figure 5D) of NRAS.

### Nras Expression is Deregulated in Animals with a Cassette Inserted Upstream of the Promoter

**Figure 6:**
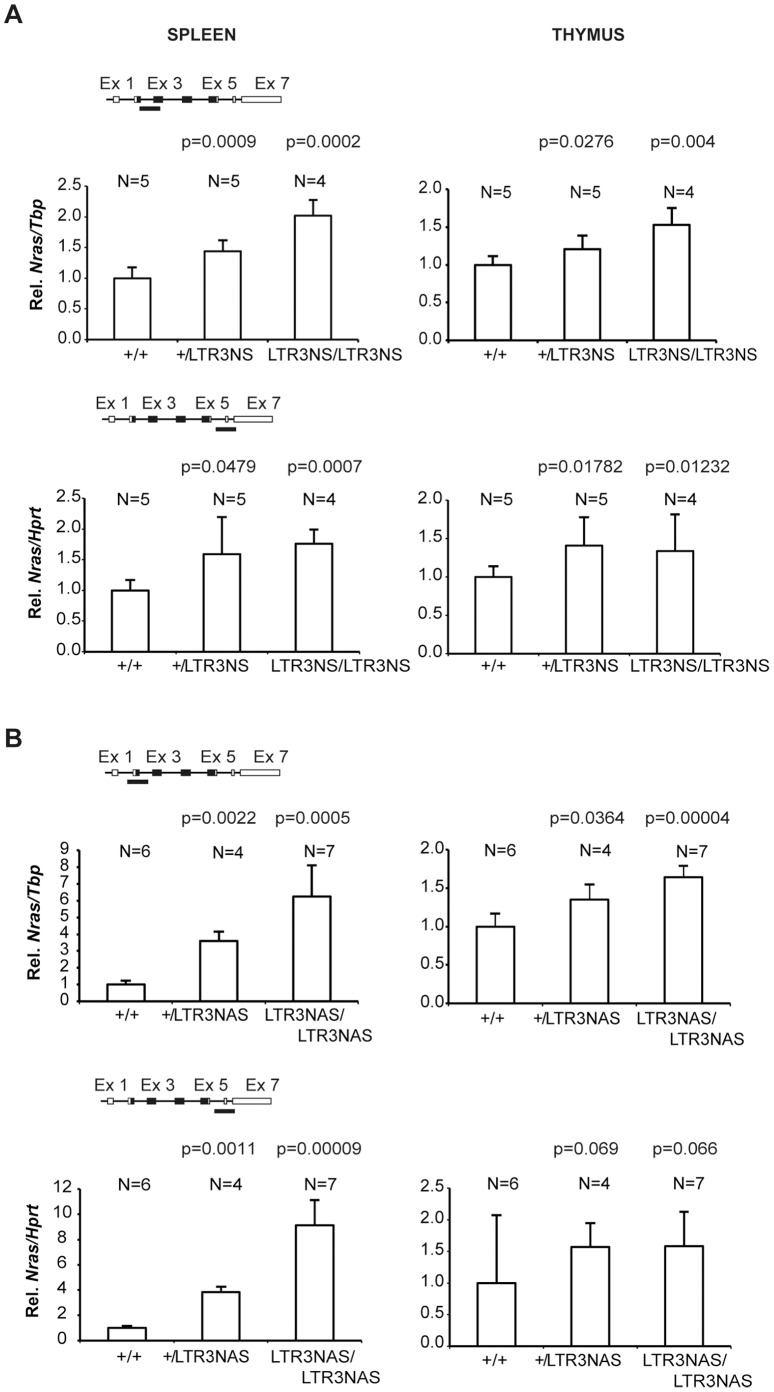
Analysis of knock-in animals harboring the LTR inserted at position 3. *Nras* expression was quantified by qPCR employing an amplicon employing two different methods, SYBR green (amplicon covering part of exon 2 and 3) or a TaqMan hydrolysis probe (amplicon covering part of exon 6 and 7). Expression was normalized to that of *Tbp* or *Hprt* depending on the employed strategy (SYBR green or TaqMan probe, respectively) and represented as relative to that of wild type animals. N represents the number of animals in the different groups. Alleles with the cassette in sense (panel A) or antisense (panel B) orientation were analyzed. Paired Student’s t test was used to determine p-values relative to +/+ animals.

To analyze the effect of insertion of an LTR upstream of the *Nras* promoter, we investigated tissues of adult animals heterozygous or homozygous for LTR3NS and LTR3NAS. These animals were phenotypically normal. We used the amplicon spanning exons 2 and 3 previously shown to correlate with protein levels as well as the amplicon spanning exons 6 and 7. The data (Figure 6) show that *Nras* expression is increased regardless of the orientation of the cassette, that heterozygous animals are intermediate between wt and homozygous knock-in animals, and that the LTR3NAS allele gives higher *Nras* expression than the LTR3NS allele. The two amplicons gave similar results. Hence, neither the LTR3NAS locus nor the LTR3NS locus cause significant activation of the cryptic promoter at the intron 3-exon 4 boundary as did LTR9NAS and LTR9AS. Since the PGK/Tn5 cassette in these strains is located further upstream from the *Nras* promoter, we did not investigate the effect of Cre-mediated cassette excision upon *Nras* expression.

## Discussion

To address how retroviral insertional mutagenesis in the germ line or in somatic tissues may deregulate host genes and cause disease we have generated a series of novel mouse strains which harbor an LTR inserted at the *Nras* locus at positions previously identified as targets for retroviral insertions in B-cell lymphomas [7]. None of the knock-in alleles cause embryonic lethality neither as homozygotes or heterozygotes. However, mice homozygous for the allele causing the highest over-expression of *Nras* in the spleen, manifest with a phenotype of granulocytosis, T-cell expansion, and decease within three weeks after birth [9].

The knock-in alleles showed deregulation of *Nras* ranging from more than ten-fold upregulation to a downregulation of three fold. Expression levels in heterozygotes were intermediates between wild type and homozygous knock-in animals. In spleen, the order of expression of mRNA including the coding exons of *Nras* among the different alleles was: LTR9S>LTR3NAS>LTR9NS>LTR3NS>LTR9AS>wt>LTR9NAS. The values observed in adult tissues roughly corresponded to those of the engineered embryonic stem cells used to generate the mouse lines, when considering that the ES cells are heterozygous for the knock-in allele. In the present study as well as in a recent publication [9], we have used the knock-in alleles for constitutive deregulation only. However, since we observed an increased level of *Nras* mRNA in adult tissues following germ-line excision of the PGK/neo, the alleles can also be used to address questions of the effect of tissue-specific or induced over-expression of wt *Nras*. A number of tools for tissue specific or inducible activation of Cre recombinase can be used for such studies [12]–[13].

For position 3, upstream of the *Nras* promoter, both cassette orientations gave rise to an increase in *Nras* expression, however, the antisense orientation to a higher level than did the sense orientation, originally detected in the B-cell lymphoma [7]. The antisense orientation upstream of a promoter is a configuration of insertional mutagenesis most commonly found for lymphoma induction by MLVs. However, in the present case the LTR is located close to the promoter, which might explain the upregulation observed by the LTR3NS as well.

For position 9, on the other hand, the cassette in sense orientation (LTR9NS) stimulated expression of *Nras* mRNA whereas the antisense orientation LTR9NAS reduced it. In case of LTR9NS we detected the normal *Nras* mRNA in which the LTR was excised as part of intron 1 as well as two types of LTR initiated mRNAs lacking exon 1 of *Nras*. The two LTR-initiated mRNAs corresponded to those observed in the original tumor 9 harboring a provirus at this position, indicating that the inserted solo-LTR functions similarly to the inserted provirus. LTR9NAS gave rise to RNA species initiated at several sites at the locus, including an antisense promoter in the LTR [8] as well as the normal *Nras* promoter and a cryptic promoter at the intron 3/exon 4 boundary of *Nras*. The enhancer of the inserted LTR activated transcription start sites in a window of about 250 bp at the cryptic promoter whereas the transcription start sites at the normal promoter are confined to a much smaller window irrespective of the presence or absence of the LTR cassette. Such enhancer activation of cryptic promoters has previously been reported to use scattered transcription start sites [14].

Endogenous retroviruses are known to be targets for epigenetic silencing of transcription in the early embryo and mouse retroviruses transferred to embryonic stem cells may be subject to such silencing mechanisms [15]. In humans, failure to sustain such silencing in adult tissues has been linked to LTR-driven expression of a neighboring gene as an oncogenic mechanism [6]. A major target for silencing of murine leukemia viruses such as Akv is overlapping with the primer binding site for proline tRNA [16], but there is also evidence of other determinants in the viral genome including the LTR [17]. In the present study the knock-in cassette contains only the LTR, and not the proline primer binding site. Only one of the knock-in alleles, LTR9NAS did result in reduced *Nras* expression. However, this reduction was only 2–3 fold and therefore relatively minor compared to the strong repression observed for some endogenous retrovirus. Whether this reduction involves epigenetic mechanisms or altered promoter configurations is not clear. We note, however, that Cre-mediated excision of the PGK/neo cassette, previously found to downregulate gene expression [11] causes upregulation of *Nras* mRNA relative to wt, suggesting that the LTR does not contribute to the reduction of expression of *Nras* mRNA in LTR9NAS. Moreover, a cryptic promoter further downstream in *Nras* is induced in LTR9NAS as well as in LTR9AS indicating that the LTR does not cause a general reduction in transcriptional activity of the target locus. The results therefore indicate that the downregulation observed in LTR9AS is unrelated to epigenetic repression targeted to the LTR.

In conclusion, we have shown that a gammaretroviral LTR inserted into the mouse germ line is transcriptionally active and mimics a number of features of retroviral insertional mutagenesis in somatic tissues such as promoter insertion, alternative splicing, enhancer insertion, activation of a cryptic promoter [18] [8] [19], and the formation of chimeric RNA initiated at retroviral antisense promoters [8]. This type of knock-in mice provides novel models for the analysis of phenotypic consequences of deregulation of target genes for retroviral insertional mutagenesis [9].

## Materials and Methods

### Knock-in, ES Cells, Animals

Homology arms for the targeting vectors were retrieved by recombineering in bacteria [20]. Linearized targeting vector DNA was electroporated into CJ7 ES cells [21]. Successful targeting was verified by Southern blot and positive ES cell clones were injected into B6D2F2 blastocysts [22]. Chimeric mice were mated with C57Bl/6J, offspring was genotyped by PCR with primers flanking the individual insertion sites. In ES cells, the PGK-TN5-neo cassette was removed by transient transfection with an expression vector coding for Cre recombinase. In mice, the PGK-TN5-neo cassette was removed by mating knock-in mice with transgenic mice expressing Cre recombinase under the control of the EIIa promoter [23].

### RNA Isolation and cDNA Synthesis

RNA was isolated from frozen tissues or cultured cells with the TRIzol Reagent (Invitrogen) using the protocol provided by the manufacturer. Random primers were used to reverse-transcribe 2.5 µg RNA of each RNA sample following the recommendations included in the Fermentas cDNA synthesis kit or the M-MLV reverse transcriptase kit (Invitrogen).

### Polymerase Chain Reaction

All reagents employed in the PCR reactions were purchased from Invitrogen except primers, which were acquired from DNA Technology. The PCR reaction mix commonly used consists of the following solutions: 5 µL 10x buffer; 8 µL 1.25 mM dNTP (deoxynucleoside triphosphate mix); 1.5 µL 50 mM MgCl_2;_ 1.5 µL forward primer (10 pmol/µL); 1.5 µL reverse primer (10 pmol/µL); 0,25 µL Taq polymerase (5 U/µL); 31.25 µL ddH_2_0; 1.5 µL template (100–500 ng).

### Quantitative-real time-PCR

qPCR analyses were performed in the Stratagene Mx3005pTM Real-time PCR machine. Two standard curves, one for the *Nras* and the other for the reference gene, composed by serial dilutions of cDNA from “wild type” tissue (spleen, thymus or liver) were included in each run. In order to determine an adequate reference gene, a pilot experiment with a few samples was conducted in which *Nras* expression was normalized against several house keeping genes. *Tbp* (TATA-box binding protein), *Gapdh*, and *Hprt* all produced equivalent results.

For the N-terminal detection the *Nras* (Mm00477878_g1) taqman probe was used with the reference *Gapdh* (4352932E) or *Hprt* (Mm00446968_m1) probes used as internal standard. C-terminal detection of *Nras* was done with Platinum SYBR Green qPCR SuperMix-UDG (Invitrogen) with primers for *Nras*:

[5' - ACTGGTCTCTCATGGCACTGTACT - 3'];

[5' - TACAAACTGGTGGTGGTTGGAGCA - 3'] and primers for *Tbp*:

[5' -AGAGAGCCACGGACAACTG - 3'];

[5' - ACTCTAGCATATTTTCTTGCTGCT - 3']

### Rapid Amplification of cDNA Ends

Initiation sites of alternative transcripts within the *Nras* gene or viral LTR were identified by the usage of the GeneRacerTM kit (Invitrogen). The sequential 5′ dephosphorylation/decapping steps included in this kit ensure the ligation of a specific adaptor RNA oligonucleotide only to full-length (previously capped) mRNA, validating the identified sequences as putative initiation site and not artifacts originated by RNA truncation. cDNA synthesis was performed following the manufacturer’s recommendations from 2 µg of RNA and utilizing the random primers provided in the kit. PCR products amplified with a DNA oligonucleotide complementary to the adaptor oligonucleotide and a gene specific primer were subsequently TOPO cloned (TOPO® TA Cloning® Kit for Sequencing, Invitrogen) in order to detect both common and rare initiation sites.

### Protein Extraction and Western Blot Analyses

Proteins were extracted from tissue or cultivated cells cultures by homogenization in RIPA buffer (10 mM Tris-HCl (pH 8.0), 150 mM NaCl, 1% Triton X-100, 0.1% SDS, 0.5% sodium deoxycholate) supplemented with proteinase inhibitors (0.2 mM PMSF, 20 µg/mL aprotinin). For detecting NRAS, 15–20 µg protein per lane was electrophoresed through 12.5% or 16% polyacrylamide gels and immunodetected with monoclonal anti-NRAS antibody (dilution 1∶300, sc-31; Santa Cruz Biotechnology) followed by visualization using the ECL Plus Western Blotting Detection system (GE Healthcare) and medical films (Konica Minolta Medical and Graphic Inc.). To confirm equal loading, membranes were stripped and re-hybridized with either an anti-GAPDH antibody (dilution 1∶300, sc-20357) or an anti-Beta-actin antibody (dilution 1∶300, sc-1616, Santa Cruz Biotechnology).
